# Analysis of a Real-World Population Participating in a Cardiac Rehabilitation Program: Cognitive Impairment, Functional Capacity, and Therapy Titration

**DOI:** 10.3390/jcm15041413

**Published:** 2026-02-11

**Authors:** Nicola Virtuoso, Francesca Palmieri, Francesco Loria, Antonio Squillante, Carmine Izzo, Martino Fortunato, Floriana Fiorentino, Emilio Sparano, Alessandro De Luca, Ilaria Fucile, Costantino Mancusi, Ornella Ferrigno, Cristina Gatto, Maria Rosaria Rusciano, D. William Molloy, Guido Iaccarino, Albino Carrizzo, Giorgia Bruno, Carmine Vecchione, Michele Ciccarelli, Valeria Visco

**Affiliations:** 1 Cardiology Unit, University Hospital “San Giovanni di Dio e Ruggi d’Aragona”, 84131 Salerno, Italy; n1virtuoso@gmail.com (N.V.); carmine.izzo93@gmail.com (C.I.); dottor.martinofortunato@gmail.com (M.F.); ornella.ferrigno@sangiovannieruggi.it (O.F.); 2 Department of Medicine, Surgery, and Dentistry, University of Salerno, 84081 Salerno, Italy; frapalmieri@unisa.it (F.P.); crgatto@unisa.it (C.G.); mrusciano@unisa.it (M.R.R.); acarrizzo@unisa.it (A.C.); gibruno@unisa.it (G.B.); cvecchione@unisa.it (C.V.); vvisco@unisa.it (V.V.); 3 Cardiac Rehabilitation Unit, University Hospital “San Giovanni di Dio e Ruggi d’Aragona”, 84131 Salerno, Italy; francescoloria94@gmail.com (F.L.); a.squillante92@gmail.com (A.S.); 4 Department of Advanced Biomedical Sciences, “Federico II” University, 80131 Naples, Italy; fucile.ilaria@gmail.com (I.F.); costantino.mancusi@unina.it (C.M.); 5 Centre for Gerontology and Rehabilitation, University College Cork, St Finbarr’s Hospital, T12 XH60 Cork City, Ireland; w.molloy@ucc.ie; 6 Department of Geriatric Medicine, Mercy University Hospital, T12 XH60 Cork City, Ireland; 7 Department of Clinical Medicine and Surgery, “Federico II” University, 80131 Naples, Italy; guiaccar@unina.it; 8 Vascular Physiopathology Unit, IRCCS Neuromed, 86077 Pozzilli, Italy

**Keywords:** cardiac rehabilitation, cardiovascular prevention, exercise therapy, cardiopulmonary exercise test, therapy titration

## Abstract

**Background:** Cardiac rehabilitation (CR) is a fundamental pillar in the therapeutic pathway of patients with cardiovascular disease, contributing significantly to improving quality of life and reducing the risk of cardiovascular event recurrence. Over the past decades, this approach has progressively evolved, integrating multidisciplinary strategies based on scientific evidence. This study aimed to conduct a detailed analysis of the anthropometric, clinical, and functional characteristics of patients enrolled in the CR Unit of the San Giovanni di Dio and Ruggi D’Aragona University Hospital in Salerno, with particular attention to therapeutic changes, drug titration, and cognitive assessment. **Methods:** Specifically, the anthropometric, clinical, laboratory, and instrumental data of 95 patients (age 66.56 ± 0.99 years, 75% male) who underwent the CR program between 2023 and 2025 were analyzed. **Results:** Patients with various diagnoses were enrolled in the CR program: 17% heart failure, 18% cardiac surgery, 20% acute coronary syndrome, 16% chronic coronary syndrome, 29% dyspnea. The patients had numerous comorbidities and risk factors: 73% arterial hypertension, 77% dyslipidemia, 35% diabetes mellitus, 33% smoking, 13% thyroid dysfunction, 47% CAD, 18% CKD, 16% COPD. At baseline, Cardiopulmonary exercise test (CPET) showed a moderately reduced functional capacity (VO_2_ peak pre-CR: 14.29 ± 0.53 mL/min/kg; VO_2_% predicted pre-CR: 62.19 ± 2.43%), and a significant improvement was recorded in meters at 6 min walk test (6MWT) post-CR (pre-CR: 306.02 ± 9.93 m vs. post-CR: 378.88 ± 13.37 m; *p* < 0.05). Notably, 22% of patients had a Qmci score < 49.4 points, indicating an MCI. Finally, the cardiovascular therapy was titrated and adapted; specifically, we recorded a significant increase in the use of SGLT2i therapy (pre-RC 22.00% vs. post-RC 34.00%; *p* < 0.05). **Conclusions:** In conclusion, CR proved to be safe and effective in enrolled patients; further studies will be needed to investigate the therapeutic modifications implemented during CR programs in more detail.

## 1. Introduction

Cardiac rehabilitation (CR) reduces mortality rates and improves the quality of life of patients with cardiovascular disease, and its use is supported by numerous clinical practice guidelines [1,2]. Indeed, participation in a medically supervised, structured, comprehensive, multidisciplinary CR program for patients after atherosclerotic cardiovascular disease events and/or revascularization, and for HF patients, is recommended by the ESC guideline in class I A [3].

CR programs can be situated in a hospital or a physician’s office, and must be supervised by a physician who is immediately available to provide consultation at all times during CR service provision. Programs must include physician-prescribed exercise, cardiac risk factor modification, psychosocial assessment, and outcomes assessment [4]. Precisely, CR programs consist of a multidisciplinary team of clinicians, including physicians, nurses, clinical exercise physiologists, behavioral health experts, dietitians, physical and respiratory therapists, and others, who collaborate [5,6,7]. Each patient must have an individualized treatment plan signed by a physician and updated every 30 days [4].

CR programs consist of three different phases: (I) the clinical phase and early mobilization at the patient’s bedside; (II) outpatient CR; (III) postcardiac rehabilitation, where patients receive guidance on maintaining an active lifestyle and continuing exercise. Precisely, the initial assessment before starting the CR program should include evaluation of cardiovascular risk profile and risk factors, investigation of comorbidities and symptoms, physical examination, electrocardiogram, biological tests, echocardiography, physical activity level, and cardiopulmonary exercise testing (CPET), to assess exercise capacity, determine maximal oxygen uptake (VO_2_ peak), and ventilatory thresholds [8]. However, an assessment of the cognitive profile is not foreseen by guidelines.

Given its broad nature and patient-centered focus, CR has the potential to enhance other aspects of disease management, including medication optimization [9]. Because most CR programs target admission shortly after a cardiac event, when variations in cardiac therapy are common, CR provides an ideal platform for significant medication management interventions [10].

However, despite the benefits now widely demonstrated, to date, only a low percentage of patients with indications adhere to CR programs [11], and real-world studies remain few. Moreover, to date, the literature lacks real-world data on titrating and implementing optimal medical therapy during the CR pathway.

Consequently, this study aims to conduct a detailed and systematic analysis of the anthropometric, clinical, and functional characteristics of patients enrolled in the CR program of our University Hospital, with particular attention to therapeutic changes, drug titration, and cognitive assessment.

## 2. Materials and Methods

We conducted a single-center, observational study, enrolling 95 patients who underwent the CR program (72% in the day-hospital and 28% in the hospital) at the Heart Failure and Cardiac Rehabilitation Unit of San Giovanni di Dio and Ruggi d’Aragona University Hospital of Salerno from January 2024 to July 2025

To be included in the study, participants had to meet the following criteria:

- Age ≥ 18 years;
- Indication to undertake a CR path, according to the latest guidelines;
- Patients able to follow a CR program.

The exclusion criteria considered were as follows:

- Pregnant women;
- Age over 80 years;
- The presence of significant neurological and/or psychiatric disorders (including epilepsy, Parkinson’s disease, stroke, bipolar disorder, psychosis, and major depressive disorder);
- History of alcohol or substance abuse;
- Use of medications with known significant effects on cognition (e.g., anti-psychotics).

Precisely, at baseline, we collected demographic and anthropometric information, medical history, vital signs, 12-lead electrocardiogram, 6 min walk test (6MWT), CPET, standard laboratory data, pharmacological treatment information, and echocardiographic parameters.

At the end of the CR program (median time: 58.31 ± 5.59 days), we recorded anthropometric information, vital signs, 12-lead electrocardiogram, 6MWT, standard laboratory data, pharmacological treatment information, echocardiographic parameters, and CPET data.

Moreover, at enrollment and at the end of the CR program, patients were administered some questionnaires to evaluate functional capacity/quality of life, cognitive abilities, and nutritional status: Italian adaptation of the EuroQol Group (EQ-5D), Quick mild cognitive impairment screen (Qmci), and Mini Nutritional Assessment (MNA).

Informed consent was obtained from all subjects in accordance with the regulations on privacy and good clinical practice.

The study protocol complies with the Declaration of Helsinki and Good Clinical Practice (GCP) guidelines and has been approved by the Campania 2 Regional Ethics Committee (Approval Number: 185,867 of 29 September 2023).

### 2.1. Laboratory Analysis

Venous blood samples were collected in the morning after an overnight fast, as a general rule. Blood chemistry was evaluated by trained personnel in our hospital laboratory using standardized methods. Triglycerides, total cholesterol (T-CHOL), and high-density lipoprotein (HDL) cholesterol were assayed enzymatically, while low-density lipoprotein (LDL) cholesterol was calculated with the formula of Friedewald. Serum glucose was measured using the glucose oxidase method. The estimated glomerular filtration rate (eGFR) was calculated by the CKD-EPI equation. The presence of chronic kidney disease (CKD) was defined by a glomerular filtration rate of less than 60 mL/min per 1.73 m^2^ (using the last available serum creatinine value at the time of enrolment).

### 2.2. Cardiopulmonary Exercise Testing

All CPETs were conducted on a cycle ergometer (Quark CPET, COSMED, Italy), with the pedaling rate set to 60 rpm. A ramp exercise protocol was systematically followed, with the workload starting at 10 watts for a warm-up period of 2 min and increasing by 6 watts every 60 s thereafter. The patients were encouraged to exercise until they felt unable to continue due to dyspnea or fatigue. The peak VO_2_ was calculated as the highest 30 s average over the final minute of the exercise, as recommended by Mezzani et al. [12]. To assess the ventilatory efficiency, we calculated the relationship between minute ventilation and carbon dioxide production (VE/VCO_2_ slope) over the entire exercise duration, as previously recommended [13].

The percentage of predicted VO_2_ denoted the achieved peak VO_2_ adjusted for age, weight, and height and expressed as a percentage. We used the equations by Wasserman and Hansen to measure the percentage of predicted VO_2_ [14].

### 2.3. Echocardiographic Measurements

To perform echocardiographic examinations, a 3.5 MHz monoplane ultrasound probe of Vivid E-9 (GE-Vingmed Ultrasound, Horten, Norway) was used, following international guidelines [15]. To avoid bias, two expert operators blinded to clinical data assessed all the parameters offline. Left ventricular ejection fraction (LVEF) was calculated by the Simpson biplane method according to the following formula: LVEF = [left ventricular end-diastolic volume (LVEDV) − LV end-systolic volume (LVESV)]/LVEDV × 100 as the mean of two measures in four and two apical chambers. A biplane method was used for left atrial volume (LAV) assessment, as well. For the evaluation of early-diastolic filling (E), in the apical long-axis view, the pulsed-wave Doppler sample volume was placed at the extremity of the tenting area of the mitral valve. In the apical 4-chamber view using Tissue Doppler Imaging (TDI), mean e’ was assessed in the basal inferoseptal and lateral LV region. Consequently, the ratio of mitral E peak velocity and averaged e’ velocity (E/e’) was calculated. Moreover, by sampling the systolic trans-tricuspid pressure gradient, calculated by the modified Bernoulli equation, the tricuspid regurgitant jet velocity was determined. Then, the systolic pulmonary artery pressure (sPAP) was calculated from the sum of the tricuspid regurgitant jet velocity and the estimated right atrial pressure, according to the inferior vena cava dimension and inspiratory collapsibility. Finally, in the apical four-chamber view, by aligning the M-mode linear cursor to the lateral tricuspid annulus and assessing the tricuspid annular plane systolic excursion (TAPSE), the right ventricular function was determined. Tricuspid annular S′ velocity (RVs′) was measured using pulsed-wave tissue Doppler.

### 2.4. Questionnaires

Firstly, patients completed the Italian version of the Qmci screen, which was created from the ABCS 135, by reweighting the original subtests and adding a logical memory section. Precisely, the Qmci is divided into six subtests, i.e., orientation, clock drawing, verbal fluency, and three tests of memory (five-word immediate and delayed recall and logical memory-immediate verbal recall of a short story), with scores from 0 (indicating severe impairment) to 100 (indicating higher levels of normal cognition) [16]. It has been reported that the Qmci is a useful test for mild cognitive impairment (MCI) and is useful in clinical practice. Indeed, we used the Qmci test because of its higher sensitivity compared with the standardized Mini-Mental State Examination (MMSE) and ABCS 135 in differentiating MCI from normal cognition [16]. The Qmci screen has shown good psychometric properties and is suitable for distinguishing normal people from subjects with MCI, as well as from patients with dementia, compared with the Montreal Cognitive Assessment (MoCA) [17], the ADAS-Cog, and the CDR [18]. These properties have been confirmed in several external validation studies performed in Italy and in other countries [19,20,21].

Every single domain of the Qmci was corrected for age and education, and an MCI condition was defined by a total Qmci corrected for age and education < 49.4, while a score < 20 is the cut-off for dementia, according to the recent literature [21].

Health-related quality of life (HRQoL) was assessed using the EQ-5D. Finally, enrolled subjects completed the MNA test, for the evaluation of nutritional status (range 0–30 points; score 24–30: normal nutritional status; score 17–23.5: patients at risk of malnutrition; score < 17: protein-calorie malnutrition).

### 2.5. Exercise Prescription

Exercise prescription followed the FITT model: Frequency, Intensity, Time, and Type of exercise. Specifically, aerobic training was prescribed 5 days per week, at moderate intensity. Each session initially lasted 20–30 min and gradually increased to 45–60 min. Aerobic activity includes treadmill walking and stationary cycling. Resistance training was associated with aerobic exercise to improve muscle strength and endurance, vital for countering lean body mass loss. They included elastic bands and cuff weights, targeting major muscle groups at 40–60% of 1-repetition maximum (1RM), according to guidelines [3]. Moreover, to prevent falls, we combined aerobic and muscle-strengthening activities with balance exercises, including static and dynamic balance training (e.g., standing on a narrow base of support and stand-and-reach). Obviously, flexibility training was incorporated into the warm-up or cool-down to prepare muscles, improve range of motion, and reduce injury risk. Finally, during the CR program, inspiratory muscle training was also performed to strengthen the diaphragm using resistive breathing devices; this exercise helped restore ventilatory function and improve exercise capacity in Phase I/II cardiac rehabilitation.

The training progression was individualized, with gradual increases in duration and intensity based on tolerance and symptoms.

## 3. Results

### 3.1. Clinical Parameters

The baseline characteristics of the study population are reported in Table 1.

The median age was 66.56 ± 0.99 years, and most patients were males (71, 74.74%). Regarding the diagnosis at the time of admission to our CR program, the following percentages were recorded: 17% (16 patients) heart failure, 20% (19 patients) acute coronary syndrome, 16% (15 patients) chronic coronary syndrome. Furthermore, 18% of patients accessed the program following cardiac surgery, and 29% for dyspnea.

Moreover, patients had a high burden of comorbidities and CVD risk factors. Indeed, hypertension was reported in 73% of cases, dyslipidemia in 77%, smoking in 33%, diabetes mellitus in 35%, history of CAD in 47%, COPD in 16%, CKD in 18%, and thyroid dysfunction in 13%. At the time of recruitment, almost all of the patients were in NYHA class I (45.26%), II (43.16%), or III (11.58%).

Overall, following the CR program, no significant changes in blood pressure parameters, heart rate, or BSA were observed (Table 1).

### 3.2. Therapy Optimization

**Figure 1:**
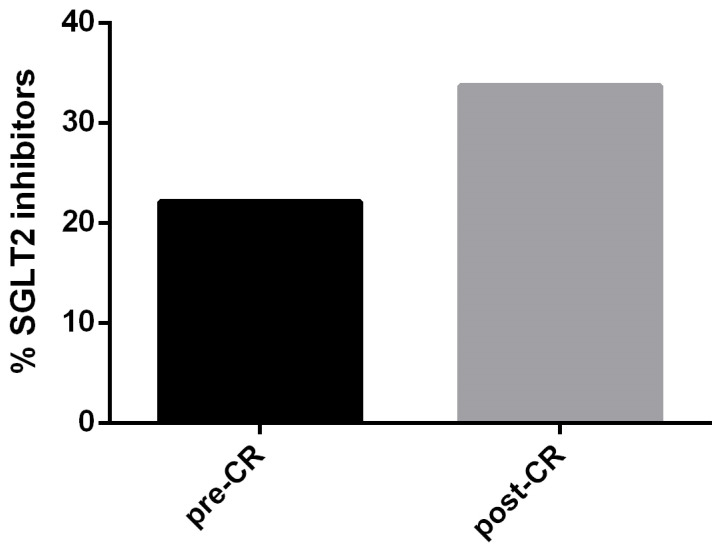
Increased use of SGLT2 inhibitors in therapy during the CR program (*p* = 0.005).

During the CR program, therapy was implemented and optimized. In particular, a statistically significant increase in the use of SGLT2 inhibitors emerged (pre-CR 22.11% vs. post-CR 33.68%, *p* = 0.005) (Figure 1).

Moreover, a trend towards an increase in the use of ARNIs (pre-CR 29.47% vs. post-CR 34.74%, *p* = 0.248) and ezetimibe (pre-CR 53.68% vs. post-CR 60.00%, *p* = 0.205) and a descalation of loop diuretic therapy (pre-CR 32.63% vs. post-CR 27.32%, *p* = 0.262) also emerges, although not statistically significant (Table 1). Precisely, lipid-lowering therapy was optimized in 7.69% of patients, while HF therapy was implemented in 14.81% of HF patients.

### 3.3. Laboratoristic and Echocardiographic Parameters

Table 2 presents the laboratory parameters: hemoglobin, glycemic and lipid profiles, renal function, and BNP (pre-CR vs. post-CR). Precisely, the analysis showed a reduction in lipid profile values (LDL: 65.14 ± 3.84 mg/dL vs. 58.22 ± 4.77 mg/dL, *p* = 0.29), but this did not reach statistical significance. Comparison of the echocardiographic parameters (Table 3) shows no significant improvements.

### 3.4. Functional Parameters and CPET

**Figure 2:**
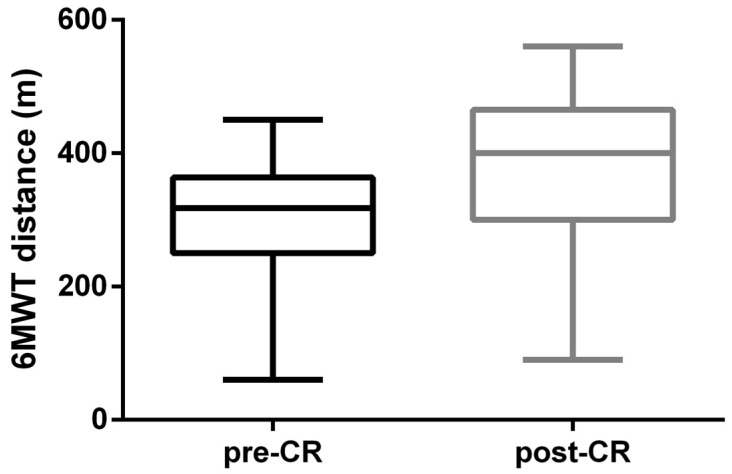
Statistically significant improvement in meters covered at the 6MWT pre-CR vs. post-CR (*p* < 0.0001).

At the 6MWT, a significant improvement in meters covered was recorded (pre-CR 306.02 ± 9.93 m vs. post-CR 378.88 ± 13.37 m; *p* < 0.0001) (Figure 2). At baseline CPET (Table 4), patients showed moderately reduced functional capacity (VO_2_ peak: 14.29 ± 0.53 mL/min/kg; VO_2_% predicted: 62.19 ± 2.43) (Table 4).

Currently, a pre- and post-RC comparison of CPET parameters has been performed only for 19 patients (age 65.10 ± 3.36), due to the lack of all follow-up data (Table 5). Precisely, the analysis showed a statistically significant improvement in maximum workload carried out (pre-CR 78.05 ± 5.44 vs. post-CR 94.89 ± 5.94 watts, *p* = 0.044), VO_2_ peak (1129.74 ± 87.19 vs. 1362.74 ± 78.87 mL/min, *p* = 0.049), VO_2_ peak/weight (14.14 ± 0.97 vs. 16.95 ± 0.85, *p* = 0.036), and circulatory power (2221.68 ± 149.46 vs. 2689.37 ± 140.94, *p* = 0.029).

### 3.5. Analysis of the Questionnaires

Table 6 shows the patients’ cognitive profile parameters, obtained following administration of the Qmci. At baseline, patients showed average low scores for some domains (verbal fluency and logical memory) and suboptimal scores for others (immediate recall, clock test, delayed recall). Notably, 22% of patients had a Qmci score < 49.4 points, indicating MCI. Currently, a pre- and post-CR comparison of the Qmci domains has not been performed due to the lack of all follow-up data.

Table 1 also shows the scores on the MNA questionnaire, with a mean score corresponding to the malnutrition risk status both pre- and post-CR, indicating a slight improvement toward normal nutritional status, but not reaching significance. Another finding emerging from the analysis is health perception (0–100), which shows a trend toward improvement post-CR (pre-CR 59.44 ± 3.72 vs. post-CR 67.73 ± 3.15), although this does not reach statistical significance, likely due to the still-small sample size.

## 4. Discussion

Specifically, two main findings emerge from our analysis: (1) our real-world monocentric study concerns a population with a high burden of risk factors and comorbidities, consequently, the results align with major longitudinal studies on CR; (2) we use the Qmci test for the first time in a CR population to assess MCI early, finding a prevalence of MCI of 22.10%.

Specifically, after an average period of 6–8 weeks of CR, no significant changes in anthropometric parameters (weight, height, BMI) were observed. This result is consistent with the literature, which shows that some studies document no significant changes in body weight after standard CR programs. Atti et al. [22] and Gomadam et al. [23] found a significant improvement in functional capacity but no significant change in BMI after 12 weeks, despite improvements in physical performance parameters. Ades et al. [24] underline that significant weight loss is observed only in programs that include specific interventions for weight reduction, whereas traditional CR involves clinically insignificant changes. Accordingly, in our CR program, specific nutritional interventions are currently missing; indeed, the MNA questionnaire also did not show significant improvements post-CR.

In a phase II CR cohort, Nozawa et al. [25] reported that approximately 50% of patients were malnourished or at risk, as assessed by MNA/MNA-SF, highlighting the usefulness of screening and the need for targeted dietary assessments. This picture is consistent with our sample. The stability of the MNA is plausible in the absence of a structured nutritional intervention in the program: in CR, simple physical training tends to maintain nutritional status, while significant improvements in the MNA score are observed when exercise is integrated with counseling and dietary support. From a methodological perspective, the use of the MNA in elderly patients undergoing CR is reliable and practical, Kather et al. [26] demonstrated excellent test reliability in patients undergoing CR after cardiac procedures, suggesting its adoption as a standard screening tool in this setting. Several cardiology studies show that lower scores are associated with worse outcomes (mortality, rehospitalizations), reinforcing the importance of identifying and managing risk early [27].

Similarly, blood pressure and HR values did not change substantially during CR, remaining essentially stable. The literature reports mixed results in this regard. In some populations, particularly in subjects with uncontrolled hypertension or in longer-duration or more intense programs, rehabilitative exercise can actually induce significant reductions in blood pressure [28,29]. Accordingly, the meta-analysis by Cornelissen et al. [30] showed that regular aerobic training reduces, on average, systolic and diastolic blood pressure, but that this effect is more marked in hypertensive patients and much less evident in normotensive or well-treated pharmacologically treated subjects. On the other hand, McKeever et al. [31] reported no significant changes in systolic blood pressure after a CR program. Finally, Gebremichael et al. [32] concluded that CR improves therapeutic adherence but shows non-significant changes in mean blood pressure levels, suggesting that the effect on resting blood pressure may be modest, especially in patients who are already well-treated (a context overlapping with ours).

In the present study, no statistically significant changes in the main laboratory parameters were observed. This data is consistent with the results found in the literature. Indeed, several studies document stability of metabolic parameters after standard cardiac rehabilitation programs, especially in patients already under good therapeutic control [33,34]; specifically, only programs with nutritional counseling seem to improve LDL levels [35]. Such evidence may suggest that the effect on biochemical parameters is time-dependent and influenced by the integration of dietary and behavioral interventions.

Likewise, echocardiographic parameters did not show statistically significant changes. The literature on this issue is conflicting. Some studies, particularly those with longer training durations or high-intensity training, observed improvements in left ventricular function. For instance, Wisløff et al. [36], in a trial of patients with chronic heart failure who underwent 12 weeks of high-intensity interval training (HIIT), they reported a significant increase in LVEF (+35% compared to baseline, *p* < 0.001). Differently, Haykowsky et al. [37] did not observe significant changes in LVEF or ventricular diameters after 12 weeks of moderate aerobic training in patients with chronic HF, although recording a significant increase in exercise capacity and cardiac output, highlighting that a medium-short duration program can improve functional performance without modifying echocardiographic parameters at rest.

We paid particular attention to assessing the cognitive status of our population. Cognitive impairment is a frequent condition in populations with cardiovascular disease and is associated with poor therapeutic adherence, worse self-management, and unfavorable clinical outcomes. The most affected functions are usually executive, attention, and memory, while orientation tends to remain preserved. The pathophysiology is multifactorial, involving chronic cerebral hypoperfusion, microangiopathy, systemic inflammation, and endothelial dysfunction [38,39,40]. In the context of CR, cognitive assessment is important for identifying vulnerable patients [38,39,40]. In our population, 22.1% of subjects had MCI. This pattern is consistent with the typical cognitive profile of cardiac populations, where the domains of attention, memory, and verbal fluency are more vulnerable [38,39,40]. Precisely, this is the first study to use the Qmci assessment in patients undergoing CR; indeed, the literature is based on alternative instruments, such as MoCA and MMSE [38,39,40,41]. Several trials and systematic reviews demonstrated that multi-domain CR programs are associated with significant cognitive increases and parallel improvements in physical performance (6MWT); precisely, the positive effect is more beneficial in patients with baseline impairment and in programs integrating aerobic exercise, functional training, and psychoeducational support [38,39,40,41,42]. However, we do not have data on the post-CR cognitive profile; furthermore, our CR program unfortunately does not currently include neurocognitive rehabilitation.

Also, subjective health perception (0–100 scale) in our sample increased from 59.4 ± 3.7 to 67.7 ± 3.1, although this difference did not reach statistical significance (*p* = 0.10). Although this finding is not significant, it can be interpreted as a favorable trend, suggesting that patients perceive an improvement in their health status, consistent with increased exercise tolerance. In the literature, some studies assess perceived quality of life as a secondary outcome in CR programs, showing statistically significant improvements, albeit using different instruments [43].

Certainly, the improvement in quality of life is due to the enhanced improvement in patients’ functional capacity [44,45]. Accordingly, we observed a significant improvement in functional capacity, assessed by 6MWT. The distance walked increased from 306.0 ± 9.93 m at baseline to 378.9 ± 13.37 m at the end of the program (*p* < 0.05), with a mean increase of +73 m (+23.6%). This gain is clinically relevant and confirms the protocol’s functional efficacy. The 6MWT is recognized as a sensitive measure of response to CR interventions. Accordingly, Bellet et al. [46] and Giannitsi et al. [43] reported that distance increments between +40 and +80 m are common in CR programs.

Correspondingly, baseline CPET indicated reduced aerobic performance, a phenomenon consistent with what is expected in cardiac populations entering CR programs. As expected, after the CR program, patients showed a statistically significant improvement in maximum workload carried out, VO2 peak/weight, and circulatory power. Similarly, Cahalin et al. [47] found mean baseline peak VO_2_ between ~13 and ~18 mL/kg/min (vs our study 14.29 ± 0.53 mL/kg/min). In a recent Chinese study, the guided rehabilitation group showed a significant improvement in peak VO_2_ after 3 months, consistent with the expectation of functional improvement [48]. These data therefore highlight the importance of CR in improving functional capacity and quality of life.

Finally, our study showed optimization and titration of medical therapy during the CR program; indeed, lipid-lowering therapy was optimized in 7.69% of patients, while HF therapy was implemented in 14.81% of HF patients. The rate of therapy optimization is not very high, likely because most patients (72.63%) achieved CR in day-hospital settings and were already followed in our cardiology outpatient clinics. However, a statistically significant increase in the use of SGLT2 inhibitors emerged, while a trend towards increased use of ARNIs and ezetimibe, and a descalation of loop diuretic therapy, was observed. Importantly, our study is among the few in the literature to report data on therapeutic optimization during the CR program. This aspect is crucial because CR represents a therapeutic window during which patients are reevaluated multidimensionally, enabling the initiation or intensification of pharmacological therapy under specialist supervision [10]. Consequently, CR not only improves functional capacity but also promotes the optimization of HF and secondary prevention medications, including SGLT2 inhibitors [10,49]. This result is even more important, since in 2023, the Focused Update of HF ESC Guidelines recommends SGLT2 inhibitors in class I A in patients with HF across the range of LVEF (HFrEF, HFmrEF, and HFpEF) [50]. Kashima et al. [49] analyzed a population of patients with cardiovascular disease and type 2 diabetes mellitus, demonstrating that the use of SGLT inhibitors during rehabilitation does not interfere with the program; thus, suggesting the safety of introducing or titrating these drugs in this context. De La Flor et al. [51] confirms that, in patients undergoing rehabilitation, the use of SGLT2 inhibitors is associated with reduced body weight and water volume, without significant loss of lean mass, supporting a favorable profile for the continuation and optimization of treatment during rehabilitation. Evidence on the early initiation of SGLT2 inhibitor therapy in patients with HF [52,53] suggests clinical benefit in the early post-acute phases, consistent with the strategy of initiating or increasing therapy during structured CR programs. Therefore, all evidence shows that it is safe and effective to introduce SGLT2 inhibitors into the therapy of CR patients, since by definition the patient is a hemodynamically stable patient, obviously with due precautions for intimate hygiene, in order to avoid genitourinary tract infections, especially in patients with risk factors such as diabetes, age ≥ 65 years, and obesity [54]. To summarize, the observed increase in SGLT2 inhibitor prescriptions after rehabilitation reflects a practical translation of international recommendations, in which CR becomes not only a time for functional recovery, but also for optimal implementation of evidence-based pharmacological therapy.

## 5. Future Perspectives

Although CR is recommended in class I A, recent data reveal that participation in CR programs is only around 30–40% of eligible patients in Europe [55]; this low participation rate is due to barriers inherent to both the healthcare system and the patient, and the main obstacles are certainly practical, such as transportation limitations, logistical difficulties for patients in rural areas, or scheduling issues. Consequently, in recent years, home-based CR (telerehabilitation) has been suggested as an alternative to increase participation rates [55]. However, there is little data in the literature regarding the effectiveness of cardiac telerehabilitation in titrating patient therapy and its effect on patients’ cognitive performance. A baseline cognitive screening would certainly be advisable before starting a telerehabilitation program to select the right patients for this remote service. For these reasons, our upcoming work will concentrate on extending our study to our cardiac telerehabilitation population.

## 6. Limitations

Our results should be interpreted considering some limitations. First, this study involves a relatively small sample size; however, this is a real-world study and the first to evaluate the use of Qmci in a CR context. Another limitation concerns the absence of Qmci data at the end of the CR program; nevertheless, our study was not conceived to evaluate the improvement in the cognitive profile of these patients, also because our CR program does not currently include neurocognitive rehabilitation. Finally, only a small subgroup of patients (n = 19) had both pre- and post-CR CPET data, due to logistic reasons; however, patients with follow-up CPET data did not differed from the rest of the cohort for age, comorbidity, and functional capacity, as showed from the comparison between baseline CPET of the entire population (Table 4) and the cohort of 19 patients (Table 5).

## 7. Conclusions

In conclusion, CR not only represents an intervention to improve the functional capacity of patients, but also and above all it is a therapeutic window, during which patients can benefit from improved pharmacological therapy and be reassessed multidimensionally. Specifically, assessing cognitive status allows for the identification of any vulnerabilities in this area, often present in cardiac patients, which can impact adherence, autonomy, and overall recovery. However, it would be important to evaluate both the impact of exercise on the post-CR cognitive profile and the presence of a specific neurocognitive rehabilitation domain in our CR programs, which is currently not present. Nutritional analysis helps prevent malnutrition, which is associated with a poor prognosis. Finally, these data offer a possibility for comparison in an area not yet explored in the literature.

## Figures and Tables

**Table 1:** Anthropometric and clinical parameters of the population (pre-CR vs. post-CR). We have reported in bold the statistically significant *p*-value.

Variables	PRE-CR (n = 95)		POST-CR (n = 95)		*p*
	n ± SEM	%	n ± SEM	%	
**Gender**			/	/	/
**Male**	71	74.74			
**F** **emale**	24	25.26			
**Age (y)**	66.56 ± 0.99	/	/	/	/
**Weight (kg)**	79.77 ± 1.78	/	79.57 ± 2.00	/	0.94
**Height (m)**	1.71 ± 0.01	/	1.70 ± 0.01	/	0.81
**BMI (kg/m^2^)**	27.35 ± 0.57	/	27.38 ± 0.66	/	0.97
**BSA (m^2^)**	1.89 ± 0.03	/	1.90 ± 0.02	/	0.73
**CR for HF**	16	16.84	/	/	/
**CR for ACS**	19	20.00	/	/	/
**CR post-PCI**	9	9.47	/	/	/
**CR post-CABG**	6	6.32	/	/	/
**CR for CCS**	15	15.79	/	/	/
**CR post-cardiosurgery**	17	17.89	/	/	/
**CR for dyspnea**	23	24.21	/	/	/
**Day-hospital**	69	72.63	/	/	/
**Hospitalization**	26	27.37	/	/	/
**Arterial hypertension**	69	72.6	/	/	/
**Diabetes mellitus**	33	34.74	/	/	/
**Smoking**	31	32.63	/	/	/
**Dysthyroidism**	12	12.63	/	/	/
**CAD**	45	47.36	/	/	/
**CKD**	17	17.89	/	/	/
**Dyslipidemia**	73	76.84	/	/	/
**COPD**	15	15.78	/	/	/
**Sinus rhythm**	81	85.26	86	90.53	0.137
**DBP (mmHg)**	74.72 ± 1.06	/	76.25 ± 1.19	/	0.34
**SBP (mmHg)**	122.15 ± 1.70	/	123.81 ± 1.64	/	0.49
**Pulse pressure (mmHg)**	47.42 ± 1.24	/	47.56 ± 1.31	/	0.94
**MAP (mmHg)**	90.53 ± 1.17	/	92.10 ± 1.20	/	0.35
**HR (bpm)**	67.30 ± 0.87	/	68.47 ± 0.95	/	0.36
**NYHA class I**	43	45.26	42	44.21	n.s.
**NYHA class II**	41	43.16	39	41.05	n.s.
**NYHA class III**	11	11.58	14	14.74	n.s.
**ACE-inhibitor**	22	23.15	18	18.95	0.319
**ARBs**	14	14.73	12	12.63	n.s.
**ARNI**	28	29.47	33	34.74	0.248
**Calcium antagonist**	10	10.52	9	9.47	n.s.
**Beta blocker**	69	72.63	66	69.47	n.s.
**Thiazide diuretic**	5	5.26	4	4.21	n.s.
**Loop diuretic**	31	32.63	26	27.37	0.262
**MRA**	36	37.89	35	36.84	n.s.
**Alphalytic**	9	9.47	11	11.57	n.s.
**Statin**	70	73.68	71	74.73	n.s.
**Ezetimibe**	51	53.68	57	60.00	0.205
**Cardioaspirin**	61	64.21	54	56.84	0.124
**P2Y12-inhibitor**	16	16.84	11	11.57	0.181
**SGLT2-inhibitor**	21	22.11	32	33.68	0.005
**Insulin**	12	12.63	12	12.63	n.s.
**PPI**	89	93.68	88	92.63	n.s.
**MNA**	22.39 ± 0.78	/	22.86 ± 0.64	/	0.64
**EQ-5D** **(0–100)**	59.44 ± 3.72	/	67.73 ± 3.15	/	0.10

ACE: angiotensin-converting enzyme; ACS: acute coronary syndrome; ARBs: angiotensin receptor blockers; ARNI: angiotensin receptor-neprilysin nhibitor; BMI: body mass index; BSA: body surface area; CABG: coronary artery bypass graft; CAD: coronary artery disease; CCS: chronic coronary syndromes; CKD: chronic kidney disease; COPD: chronic obstructive pulmonary disease; CR: cardiac rehabilitation; DBP: diastolic blood pressure; EQ-5D: EuroQoL group; HR: heart rate; HF: heart failure; MAP: mean arterial pressure; MNA: mini nutritional assessment; MRA: mineralcorticoid receptor antagonist; NYHA: New York Heart Association; PCI: percutaneous coronary interventions; PPI: proton pump inhibitors; SBP: systolic blood pressure; SGLT2: sodium-glucose cotransporter-2. The symbol “/”indicates that the value is equal to the baseline.

**Table 2:** Comparison of laboratory parameters of pre-CR vs. post-CR.

Variables	PRE-CR (n = 95)	POST-CR (n = 95)	*p*
	n ± SEM	n ± SEM	
**Glicemia (mg/dL)**	112.57 ± 4.97	120.70 ± 7.72	0.36
**T-CHOL (mg/dL)**	131.67 ± 4.90	125.83 ± 6.83	0.49
**HDL (mg/dL)**	45.32 ± 1.56	44.17 ± 2.24	0.67
**LDL (mg/dL)**	65.14 ± 3.84	58.22 ± 4.77	0.29
**Triglycerides (mg/dL)**	116.04 ± 17.73	113.31 ± 16.37	0.92
**Hb (g/dL)**	12.86 ± 0.21	12.93 ± 0.24	0.83
**Creatinine (mg/dL)**	1.15 ± 0.09	1.24 ± 0.14	0.58
**BUN (mg/dL)**	45.67 ± 2.01	52.65 ± 3.85	0.08
**eGFR (m** **L** **/min)**	74.46 ± 2.38	69.18 ± 3.40	0.20
**BNP (pg/mL)**	296.25 ± 64.35	362.73 ± 135.02	0.63

T-CHOL: total cholesterol; HDL: high-density lipoprotein; LDL: low-density lipoprotein; Hb: hemoglobin; eGFR: estimated glomerular filtration rate; BNP: brain natriuretic peptide; BUN: blood urea nitrogen.

**Table 3:** Comparison of echocardiographic parameters pre-CR vs. post-CR.

Variables	PRE-CR (n = 95)		POST-CR (n = 95)		*p*
	n ± SEM	%	n ± SEM	%	
**IVSd (mm)**	11.51 ± 0.25	/	11.43 ± 0.38	/	0.85
**E/e’**	10.50 ± 0.45	/	10.83 ± 0.70	/	0.68
**TAPSE (mm)**	19.31 ± 0.54	/	18.51 ± 0.51	/	0.32
**Rvs’ (cm/sec)**	9.86 ± 0.19	/	9.36 ± 0.28	/	0.12
**sPAP (mmHg)**	29.47 ± 0.76	/	29.26 ± 0.74	/	0.85
**LVEF (%)**	49.50 ± 1.27	/	46.89 ± 1.62	/	0.21
**LAVi (m** **L** **/m^2^)**	36.51 ± 0.96	/	36.89 ± 1.53	/	0.42
**LVEDVi (m** **L** **/m^2^)**	55.67 ± 2.00	/	58.90 ± 3.01	/	0.36
**LVH**	10	10.53	15	15.78	0.09

IVSd: end-diastolic intraventricular septum diameter; E: early-wave transmitral diastolic velocity; e’: early-diastolic velocity at tissue Doppler imaging; TAPSE: tricuspid annular plane systolic excursion; Rvs’: right ventricular systolic myocardial velocity at tissue Doppler imaging; sPAP: pulmonary arterial systolic pressure; LVEF: left ventricular ejection fraction; LAVi: left atrium volume index; LVEDVi: left ventricular diastolic volume index; LVH: left ventricular hypertrophy.

**Table 4:** CPET parameters.

Variables	PRE-CR (n = 95)
**Predicted VO_2_ (m** **L** **/min)**	1583.03 ± 42.91
**Watts max**	77.72 ± 4.04
**Watts threshold**	48.68 ± 3.16
**VO_2_ (mL/min) peak**	1151.42 ± 50.63
**VO_2_ peak/weight**	14.29 ± 0.53
**ideal VO_2_ peak/weight**	19.88 ± 0.49
**Predicted VO_2_%**	62.19 ± 2.43
**VO_2_ (mL/min) threshold**	859.89 ± 40.66
**VE/VCO_2_ slope**	36.97 ± 1.44
**Basal HR**	65.72 ± 2.12
**Peak HR**	107.33 ± 2.37
**% max predicted HR**	69.71 ± 1.41
**% ventilatory reserve**	49.44 ± 2.11
**Peak RQ**	1.03 ± 0.02
**O_2_% pulse**	91.48 ± 3.43
**Circulatory power**	2261.431 ± 83.75
**Ventilatory power**	4.58 ± 0.15

HR: heart rate; RQ: respiratory quotient; VO_2_: oxygen consumption; VE/VCO_2_: minute ventilation to carbon dioxide production.

**Table 5:** Comparison of CPET parameters pre- and post-CR.

Variables	PRE-CR (n = 19)	POST-CR (n = 19)	*p*
**Predicted VO_2_ (mL/min)**	1686.11 ± 72.72	1720.37 ± 74.63	0.744
**Watts max**	78.05 ± 5.44	94.89 ± 5.94	0.044
**Watts threshold**	50.89 ± 4.45	58.21 ± 5.21	0.295
**VO_2_ (mL/min) peak**	1129.74 ± 87.19	1362.74 ± 78.87	0.049
**VO_2_ peak/weight**	14.14 ± 0.97	16.95 ± 0.85	0.036
**idealVO_2_ peak/weight**	21.21 ± 0.86	21.71 ± 0.91	0.692
**Predicted VO_2_%**	57.95 ± 5.09	66.63 ± 4.19	0.196
**VO_2_ (ml/min) threshold**	876.42 ± 78.78	1012.89 ± 68.32	0.199
**VE/VCO_2_ slope**	34.89 ± 2.19	32.64 ± 2.12	0.473
**Basal HR**	60.58 ± 3.35	61.16 ± 2.31	0.888
**Peak HR**	103.53 ± 3.64	105.47 ± 4.23	0.729
**% max predicted HR**	67.96 ± 2.12	64.81 ± 3.33	0.452
**% ventilatory reserve**	53.72 ± 4.20	53.21 ± 2.51	0.918
**Peak RQ**	1.06 ± 0.03	1.03 ± 0.02	0.918
**O_2_% pulse**	89.21 ± 6.36	99.53 ± 5.19	0.217
**Circulatory power**	2221.68 ± 149.46	2689.37 ± 140.94	0.029
**Ventilatory power**	4.83 ± 0.29	5.48 ± 0.64	0.361

HR: heart rate; RQ: respiratory quotient; VO_2_: oxygen consumption; VE/VCO_2_: minute ventilation to carbon dioxide production.

**Table 6:** Qmci parameters.

Variables	PRE-CR (n = 95)	
	n ± SEM	%
**Years of education (y)**	12.69± 0.88	/
**Immediate recall**	4.18 ± 0.23	/
**Delayed recall**	11.18 ± 1.00	/
**Orientation**	9.77 ± 0.16	/
**Clock drawing**	11.81 ± 0.84	/
**Verbal fluency**	8.89 ± 0.77	/
**Logic memory**	12.00 ± 1.33	/
**Qmci total score**	57.85 ± 3.10	/
**MCI (Qmci< 49.4 points)**	21	22.10

## Data Availability

The data underlying this article will be shared on reasonable request to the corresponding author.

## Abbreviations

The following abbreviations are used in this manuscript:

6MWT	6 min walk test
ACE	angiotensin-converting enzyme
ACS	acute coronary syndrome
ARBs	angiotensin receptor blockers
ARNI	angiotensin receptor-neprilysin inhibitor
BMI	body mass index
BNP	brain natriuretic peptide
BSA	body surface area
BUN	blood urea nitrogen
CABG	coronary artery bypass graft
CAD	coronary artery disease
CCS	chronic coronary syndromes
CKD	chronic kidney disease
COPD	chronic obstructive pulmonary disease
CPET	cardiopulmonary exercise test
CR	cardiac rehabilitation
DBP	diastolic blood pressure
E	early wave transmitral diastolic velocity
e’	early diastolic velocity at tissue Doppler imaging
eGFR	estimated glomerular filtration rate
EQ-5D	euroQoL group
Hb	hemoglobin
HDL	high-density lipoprotein
HF	heart failure
HR	heart rate
IVSd	end-diastolic intraventricular septum diameter
LAV	left atrial volume
LDL	low-density lipoprotein
LVEDV	left ventricular end-diastolic volume
LVEF	left ventricular ejection fraction
LVESV	left ventricular end-systolic volume
LVH	left ventricular hypertrophy
MAP	mean arterial pressure
MCI	mild cognitive impairment
MNA	mini nutritional assessment
MRA	mineralcorticoid receptor antagonist
NYHA	New York Heart Association
PCI	per-cutaneous coronary interventions
PPI	proton pump inhibitors
Qmci	quick mild cognitive impairment
RQ	respiratory quotient
RVs’	right ventricular systolic myocardial velocity at tissue Doppler imaging
SBP	systolic blood pressure
SGLT2:	sodium-glucose cotransporter-2
sPAP	systolic pulmonary artery pressure
TAPSE	tricuspid annular plane systolic excursion
T-CHOL	total cholesterol
TDI	tissue Doppler imaging
VE/VCO_2_	minute ventilation to carbon dioxide production
VO_2_	oxygen consumption
